# Research with Black Communities to Inform Co-Development of a Framework for Anti-Racist Health and Community Programming

**DOI:** 10.1177/08445621241254883

**Published:** 2024-05-15

**Authors:** Jaimeson Canie, Selma Tobah, Anne-Marie Sanchez, C. Nadine Wathen

**Affiliations:** 1 Arthur Labatt Family School of Nursing, Western University, London, Ontario, Canada; 2 London InterCommunity Health Centre, London, Ontario, Canada

**Keywords:** antiracism, systemic racism, qualitative research, community health centres, primary care

## Abstract

**Study Background:**

The effects of systemic racism were exacerbated and amplified throughout the COVID-19 pandemic. The resurgence of the “Black Lives Matter” movement in North America brought awareness to the public, especially white people, of the impacts of systemic racism in society and the urgent need for large-scale and institutional anti-racism work.

**Purpose:**

In collaboration with a local Community Health Centre, this research focused on identifying priority areas for tailored and co-developed anti-Black racism interventions in health services and community programming, as well as examining how purposeful relationships can be created with African, Caribbean, and Black (ACB) communities in London, Ontario.

**Methods:**

Semi-structured interviews were conducted in either French or English with nine formal or informal leaders from London's ACB communities. Interpretive description methodology guided analysis and interpretation.

**Results:**

Participants indicated that anti-Black racism is ever-present in the community and in their lives, with systemic racism causing the most harm. Racism should be addressed by creating ACB-specific services, and education for non-Black communities; increased representation, inclusion, and engagement of ACB people within organizations, especially in leadership roles, are essential. A framework based on study findings to guide how organizations can develop authentic and purposeful relationships with ACB communities is presented.

**Conclusions:**

Organizations will continue to perpetuate systemic racism unless they actively seek to be anti-racist and implement strategies and policies to this end. The proposed framework can guide partnerships between health and community organizations and ACB communities, and support co-development of strategies to address anti-Black racism.

## Background and purpose

The effects of systemic racism were exacerbated and amplified throughout the COVID-19 pandemic, with higher rates of SARS-CoV-2 infection and death among racialized communities, including in Ontario (Ezezika et al., 2022). Additionally, the response to the deaths of George Floyd and Breonna Taylor in the United States, among other high-profile cases of Black people assaulted and killed by police, pushed the ‘Black Lives Matter’ movement in North America into mainstream consciousness. This movement brought awareness to the public, especially White people, of the impacts of systemic racism in society and the urgent need for large-scale and institutional anti-racism work (Bailly et al., 2021). In response to this, Ontario's Middlesex-London Health Unit (MLHU, 2020), among others, declared racism a “public health crisis”, calling on the community to recognize and address its effects. In 2021, the City of London formed the anti-racism and anti-oppression division which seeks to work with Black communities to identify and address their needs; to date, the Anti-Black Racism Plan is in its’ beginning stages, with little known about how to meet ACB communities’ needs (City of London, 2023).

Systemic racism occurs when ideas of White superiority become embedded in the policies and practices of institutions or systems, including public health systems, resulting in systematic disadvantages for racialized groups, notably Black and Indigenous People (Canadian Public Health Association, 2018; Crenshaw et al., 1996). In Canada, systemic racism against Black people is rooted in the European colonization of Africa and the transatlantic slave trade; racist ideologies established in that era continue to drive discrimination through systems and policies today (Government of Canada, 2020). It is a public health crisis that directly reduces the health and well-being of racialized communities.

Racism reduces access to the social determinants of health, and appropriate health services, placing racialized people at increased risk for poor health outcomes (Ezezika et al., 2022). Black populations in Canada face many inequities; in particular, racial discrimination at the individual and systemic level, increased poverty, immigration status, precarious housing, and underemployment affect these inequities (Olanlesi-Aliu et al., 2023). Some results of systemic anti-Black racism include racial profiling, over-policing, over-representation of Black people in the criminal justice system, over-representation of Black youth in welfare systems; discrimination in the healthcare system, and low or no representation of Black individuals in leadership positions (Government of Canada, 2020). These effects, i.e., the prolonged exposure to the trauma of racism and oppression, lead to poor health outcomes, both mental and physical (Olanlesi-Aliu et al., 2023).

The lack of Black representation in healthcare leadership, culturally appropriate services, professionals, and services available in relevant languages intersect, preventing ACB communities from accessing timely and adequate health and social services (Etowa et al., 2021). In Ontario, only 2.3% of physicians are Black, despite 4.7% of the Ontario population identifying as Black (DasGupta et al., 2020). Okeke-Inhejirika et al. (2020) found that there is a lack of culturally appropriate services for Sub-Saharan African migrants in Canada, with a significant gap existing in mental health services. Further, the effects of anti-Black racism at a systemic level lead to inadequate health and social care, resulting in disease or death that was preventable had people received better quality care (Bluthenthal, 2021; National Collaborating Centre for Determinants of Health, 2018). The COVID-19 pandemic brought to the forefront the long-standing health disparities that ACB communities face (Public Health Ontario, 2022). Black people were 1.5 times more likely to die from a COVID-19 infection compared to White people, even after adjusting for other factors and comorbidities (Thompson et al., 2021). These striking statistics led many health and social service organizations to examine their policies and practices to explore strategies to improve health outcomes for ACB communities.

This increased attention to anti-Black racism has led local, provincial, and national healthcare and social service organizations and communities to begin more seriously addressing this issue. Organizations will continue to perpetuate systemic racism until they become actively anti-racist and implement strategies and policies to achieve this (Boykin et al., 2020). For this reason, there is a need to identify Black communities’ priorities and interventions for anti-Black racism, as well as to understand how purposeful relationships can be created with these communities. This project endeavoured to do this work in London, Ontario. The city's Black population accounts for approximately 15% of visible minorities and 3.5% of the city's total population, yet there is a lack of health and social services and funding for these services specifically for African, Caribbean, and Black (ACB) communities in London (MLHU, 2021). It is also useful to understand how these needs could vary among the Black diaspora in London, which includes a diversity of long-established Black Canadians descended from Black Africans fleeing slavery in the US prior to emancipation, as well as more recent newcomers from both English and French colonized countries in the Caribbean and Africa. French is one of the most common languages spoken by Black Londoners (MLHU, 2021), meaning that we paid specific attention to issues relevant to the francophone community.

The Registered Nurses’ Association of Ontario released a report titled *Acknowledging, Addressing and Tackling Anti-Black Racism and Discrimination Within the Nursing Profession* calling for an end to anti-Black racism in the nursing profession and highlighting the need to dismantle systemic racism in Ontario (RNAO, 2022). One of the recommendations of the report included to “hold all [nurses] accountable for addressing racial discrimination and develop specific strategies to combat it”. This study serves to help nurses better understand how they can address anti-Black racism and form meaningful relationships with ACB communities.

### Research questions

This study was in partnership with London InterCommunity Health Centre (LIHC) and was conducted in the context of London, Ontario. The research questions were:

1. How can health and social service organizations form sustainable, authentic, and purposeful relationships with ACB communities that prioritize the needs (as identified by participants) of these communities?
2. What strategies are identified by ACB community leaders to address anti-Black racism?

## Methods and procedures

In conducting this study, the primary researcher, a White woman and professional, recognizes the potential influence of their own identity on the topic. Acknowledging their positionality, the researcher sought to maintain reflexivity throughout the entire research process and present a more nuanced understanding of the participants’ experiences within the broader context of systemic racism. While the team included women of varied cultural backgrounds, it did not include a member of the local Black community, leading us to attend to this issue in follow-up interviews.

Participants were at least 18 years of age, living in London-Middlesex, and self-identifying as African, Caribbean, and/or Black. They were involved in ACB community service work (e.g., as part of a formal or informal group or association, i.e., a community organization providing specific healthcare services or groups providing peer support), in London, Ontario, and were able to complete the interview in either English or French. Leaders were targeted due to their unique ability to offer perspectives of the broader ACB communities in London, Ontario. Participants were provided with an honorarium as a token of appreciation. A purposeful approach to sampling was employed. For data collection, people who fit our criteria were invited by staff from our community partner's agency via email using a pre-approved letter to participate in a videoconference interview based on their knowledge, expertise and experience working with/within ACB communities in London using a semi-structured interview guide. Snowball sampling was also employed where participants notified others who might have been eligible or interested in participating of the study. Data collection and analysis occurred from January to August 2022 using Interpretive Description (ID) methodology (Thorne, 2016). The study was broadly framed in a critical anti-racist/anti-oppressive paradigm, though ID itself, given its practice focus, does not require a theoretical orientation (Thorne, 2016). ID allowed the authors to explore the complex experiences of leaders within the ACB communities while allowing the flexibility to tailor the study design to meet the needs of the communities (Thorne, 2016); for example, conducting follow-up discussions when there was slow uptake for recruitment. ID also helped to produce findings that are practical and applicable to the practice setting in terms of policy and programs (Thorne, 2016). Participants were emailed a Letter of Information and Consent prior to the interview and before the interview commenced, were asked to provide audio-recorded verbal consent. Data collection and analysis occurred simultaneously until enough data had been collected to provide new, logical, and meaningful contributions to the phenomena and those in the field (Thorne, 2016). All interviews were transcribed verbatim by the researcher in the original language of the interview to not lose any meaning or nuances of the spoken language during translation. Two members of the research team independently developed initial codes and applied them manually to one transcript, then came together to compare their codes and develop a codebook in English. Next, one member of the research team manually applied the English code book to all the other interview transcripts, including the French transcripts. Next, two other transcripts were selected at random, and the second member of the research team verified the coding of these transcripts. After this initial coding, the “Sort and Sift, Think & Shift” method (Maietta et al., 2021) of data analysis was used to allow for the creation of themes relevant to the research questions and real-time feedback to the research partner (LIHC). After coding each interview, the author re-read transcripts and pulled quotes that caused the author to pause and reflect; these were combined to create a quotation inventory. Next, the author reflected on each quotation and its meaning regarding the research question and explored this relationship through writing. Finally, the quotation inventory and memos used in combination allowed the creation of an episode profile that recounted the story of each participant's perspective in a holistic way. Afterwards, mining, bridging, threading, and reflection tools were used to reflect and analyze the data, developing key themes (Maietta et al., 2021). All study procedures were in compliance with Western University's Non-Medical Research Ethics Board (Protocol No. 119854).

## Results

A total of nine interviews were conducted, as well as three follow-up discussions with 2 of the participants, and one staff at LIHC who was aiding with recruitment, to investigate the framing of the study as expressed by some participants, and as a form of member-checking. The interviews ranged from 30 to 90 min, averaging 58 min. All participants held either a paid or volunteer position within the health or social service sector in London, Ontario. The length of time the participants had been in that role ranged from seven months to 27 years. Participants self-identified as Black, African, Mixed Race, Ga, Caribbean, Congolese, West Indian, and Canadian, with 22.2% of participants born in Canada, and the other 77.8% having immigrated to Canada in the prior four to 41 years. Both men (44.5%) and women (55.5%) were interviewed, and ages ranged from 29 years to 65  +  years. Of the nine primary interviews, two were conducted in French and seven in English; all follow-up discussions were conducted in English. Follow-up discussions (less than 30 min) were informal and unstructured, seeking to understand the initial slow uptake of participation during recruitment.

**Figure 1.**
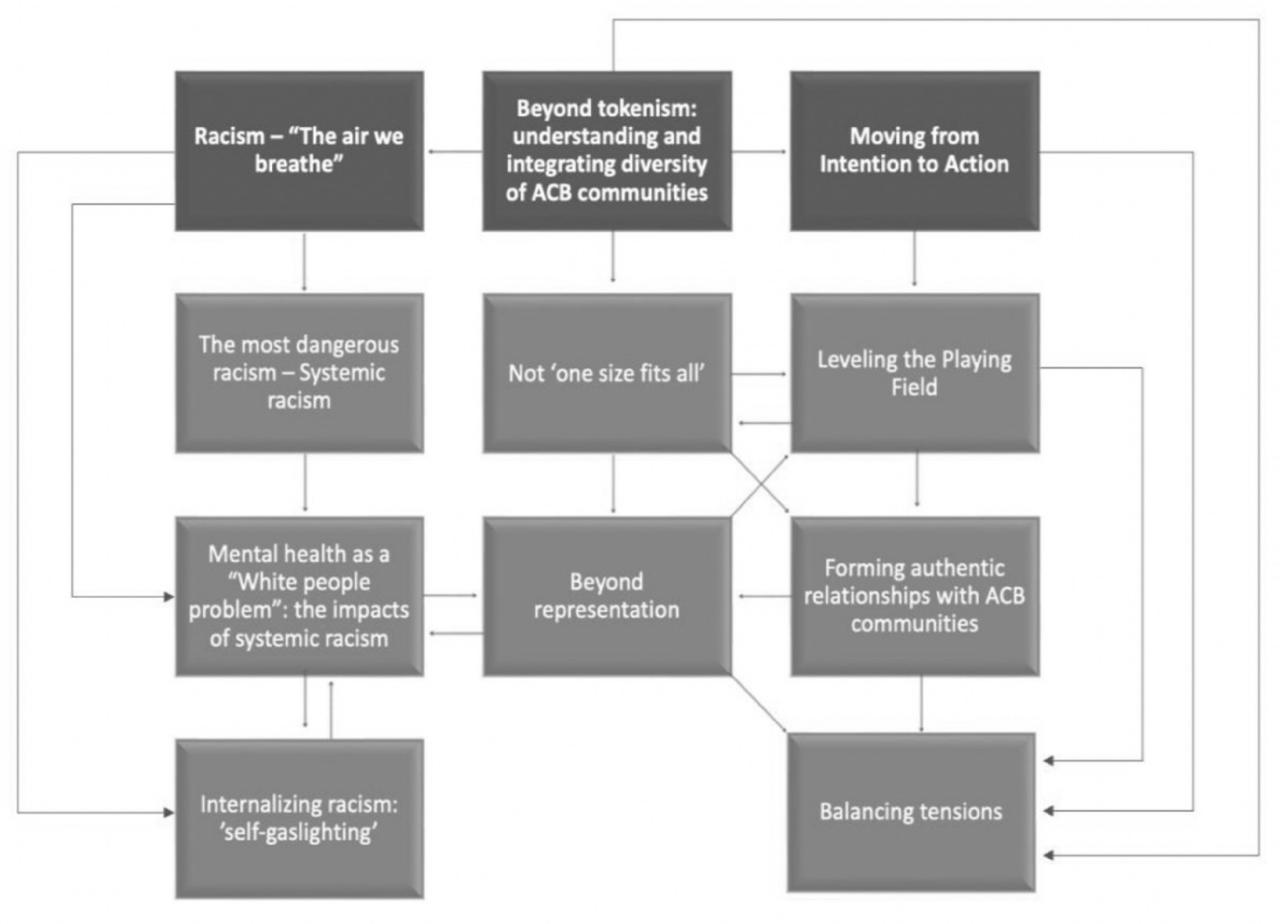
Themes, subthemes, and conceptual links among them.

Three key themes identified were: (1) Racism- “The air we breathe”; (2) Beyond tokenism: understanding and integrating diversity of ACB communities; (3) Moving from intention to action, representing the views of most, if not all participants. Figure 1 shows these, along with their subthemes, and the conceptual links among them.

### Racism – “the air we breathe”

To those who live in a Black body, in London and anywhere in Canada, racism is expected: “We talk about racism all the time, it's like the air we breathe, because if you live in a Black body, that's part of your life. It's just normal” (Participant #1). Racism is a daily experience for London's ACB communities. One participant was explicit, “Racism is real. It's real and it's here in London. It's everywhere” (Participant #1). Microaggressions are the most common and subtle type; however, systemic racism was identified as a priority to address to improve health: “The focus to me should be the systemic racism because that's the most dangerous to me” (Participant #2). Systemic racism is at play in healthcare and social services and perpetuates barriers to care. This was emphasized throughout the data; as one participant said, “I think healthcare is probably the most dangerous thing for us besides the police and just the government in general” (Participant #3) pointing to the effects of systemic racism within the healthcare system. Another reiterated this idea, “I'm not saying everybody is bad, the system is not working” and described that there is a “culture of racism within healthcare” (Participant #7). The current systems for healthcare, social services and education are built on colonialism and uphold Eurocentric values which continue to harm ACB people. Further, systemic racism perpetuates barriers for racialized people to access services: “So, the service… it's there… the need can be met, but how to ensure the colour of my skin doesn’t become a barrier to access that” (Participant #2). Participants shared that systemic racism resulted in unequal employment opportunities for qualified ACB people, unequal educational opportunities for Black children, inadequate medical treatment for Black women, the targeting of Black men by the police, higher rates of HIV among ACB communities, and overall, contributed to poor health outcomes.

An important example of how embedded racist beliefs in systems such as healthcare can play out for people is the recurring idea, expressed by participants, which is that mental health is a “white people problem” (Participant #3, Participant #6). Another participant describes the dismissal of ACB mental health issues:

La santé mentale pour les Africains, les Noirs, ce sont des sujets tabous, on ne parle pas. Tu dis à quelqu'un, tu es déprimé, il te dit, mais tu es faux. [Mental health for Africans, for Black people, are taboo subjects, we don’t talk about it. If you tell someone you are depressed, they will tell you no you’re wrong]. (Participant #9)

Another participant described the need for mental health education:

I see [ACB people] having mental health issues not addressed, because mental health issues are not really talked about in the community. It's not. It's a taboo. So that itself is a huge, huge, huge issue because they don't think about it, and we need education, where's the education? (Participant #7).

Additionally, participants noted that another barrier to health and social services for ACB community members was that they were not aware that services existed, or that if they knew about them, they may have believed that they were not tailored to their cultural needs or experiences. Participants urged that there is a need for ACB-specific services tailored to their needs to decrease barriers and improve access to safe and culturally appropriate care.

Although racism is real and ever-present, not all ACB people have the same experiences. Some are unsure of the racism that they are experiencing, and others are questioning whether it is all in their heads (Participant #3) engaging in a process called ‘self-gaslighting’. Participants described a process of minimizing or denying their experiences of racism, which can result in the internalization of the message that they are not deserving of care, again increasing the risk of adverse health events.

### Beyond tokenism: Understanding and integrating the diversity of ACB communities

Not all ACB people, or groups, have the same needs. However, participants noted that they are often seen only according to the colour of their skin, leading to generalized assumptions and harmful stereotypes. Participants were very clear on the need for authentic recognition, understanding and representation of ACB communities within organizations, especially as service providers, “the lack of representation really further marginalizes people from accessing services because they don’t feel that they’re going to be seen or heard because the staff that's working there doesn’t look like them” (Participant #3). This was especially important for mental health services:

As much as [White therapists] can give me support, there's intricacies of race and underlying connotations of what that means, especially as a Black woman that to have somebody that recognizes that and can understand that is important and humanizing of my experience. (Participant #3)

ACB-identifying service and Francophone service providers are needed to increase access to and uptake of services. To address the lack of French-speaking service providers, a solution suggested by our participants is to have a centralized French system navigator to communicate with clients, to determine their needs, and assist them with locating services. To allow for the creation of better relationships with ACB communities, it was proposed that agencies have a specific position that focuses solely on ACB health and well-being. Additionally, there needs to be representation within leadership positions:

“We have to see dually qualified Black people in leadership positions so that when we are discussing strategy and policy, it is not from people who don't live in a Black body who are trying to tell Black people what is good for them” (Participant #1).

A participant shared that racial discrimination contributes to underemployment and career advancement, suggesting a key strategy is to “level the playing field” (Participant #4) when it comes to career opportunities for those who could support tailored programs and services. This means ensuring that the conditions in which one can gain entry into employment are equitable and that organizations prioritize equity, diversity and inclusion in hiring.

### Moving from intention to action

There is a need for explicit processes to form authentic relationships with ACB communities. Authenticity was identified by many participants as the most important, and foundational, requirement; or the idea that “[researchers] are not just coming in to check boxes” (Participant #3). Power imbalances need to be recognized and levelled throughout the process. Service providers and organizations need to uncover their biases, unlearn them, and make an active effort to be anti-racist. Organizations and individuals must educate themselves: “That is a must: be educated, when people become educated, people become less fearful and when they’re less fearful, then you can build bridges” (Participant #1). Those who educate themselves about racism, its local and historical contexts prior to approaching an ACB community, are not presenting a burden, while those who come expecting to be educated by the community, pose the burden to relive often traumatic experiences. As one said: “Learn. Research. Get knowledge in order for us to even have a discussion because if you’re coming from square one… we are tired of that” (Participant #7). To meet the needs of the ACB communities, participants suggested that organizations need to be able to “work outside the box” (Participant #2). Participants recommended that all staff at every level of an organization should receive cultural safety training. ACB leaders would like to see concrete action to address anti-Black racism, and implored the system leaders “practice what you preach” (Participant #2). As stated by another, “I would really like a consistent doing action. Not just saying our policies are inclusive of this and that. Actually asking [questions]” (Participant #6). In essence, participants felt that an organization's policies were performative and counter-productive in terms of eroding trust. Finally, participants stated that asking communities for feedback is necessary in forming relationships and implementing anti-racism strategies.

### Balancing tensions

Entering this research, we were aware that a common concern is that the people affected most by racism are expected to shoulder the burden of solving this problem that they did not create (Layne et al., 2022). We wanted to avoid this, so we asked the question: “How do we make sure that potential strategies and solutions reflect the needs of ACB communities, without placing further burden on them?” In response, one participant summarized it best, pointing out the difference between “Can you help us help you?” versus “I don’t think that there is racism. Can you show me? Can you educate me?” (Participant #2).

Despite the use of the former approach (‘can you help us help you?’), an unanticipated theme that emerged during the study was a tension we believe to be related to the nuance of ‘educate yourself before coming to us’ versus ‘nothing about us without us’, referring to the balancing of the community's input/involvement in the research, without going to the community for basic education on racism. We attempted to balance this nuance throughout the research, deciding that ultimately, we needed to further investigate how we can conduct research with racialized communities in a way that decreases this tension. Engaging thoughtfully and without defensiveness with this concern allowed us to reflect on the process of conducting research with a marginalized group without membership in that group while managing power imbalances, recognizing potential risks, and mitigating any harm. To explore this further, we conducted informal follow-up conversations with leaders who had expressed these kinds of concerns.

Through these conversations, we found that many ACB community members were glad that this research was being done, but despite this feeling, the uptake for interviews was relatively slow (though ultimately reached a reasonable sample of nine participants). A key informant explained that many Black people have a “jaded” (Participant #5) view of research and are “tired” (Participant #5) of participating in research and not seeing the results translate into action benefitting the communities. To offset potential tensions and increase engagement, participants suggested that researchers involve affected communities from the beginning *before* the research officially begins, asking them their thoughts, what research needs to be done, and the best way to do it, such as by using a participatory action research approach. Participants suggested this could be achieved by having an ACB person working with the organization or research team to help connect to the community and alleviate these tensions. To address the issue that communities feel that researchers come in, extract the information they want, and never return to share results or benefit the community, informants recommended holding a forum to share and discuss the results with the community. A graphic research summary was prepared and shared with participants.

## Discussion

As reviewed above, and reiterated by participants, systemic racism leads to poor individual and population-level health outcomes (Ezezika et al., 2022; Griffith et al., 2007; Jones, 2000; Rankine, 2014), and is indeed present in Ontario's institutions and communities (Ontario Health, 2023). In our study, participants shared examples of overt racism, microaggressions and racial stereotypes that impacted their lives, but noted that systemic racism had the greatest influence on their own health and well-being, and that of their communities.

Consistent with previous literature (Griffith et al., 2007; Rankine, 2014), racism was described by our participants as the biggest barrier to healthcare for ACB people in London, due to both its embeddedness in government legislation and institutional policies, and by actively creating barriers to accessing services, both of which increase health disparities among Black people. There is a critical need for evidence-based interventions that address racism at the systemic level and inform organizations and institutions on how to form meaningful relationships with racialized groups (Came & Griffith, 2018; Hassen et al., 2021). This is especially important in healthcare organizations since racism was identified by participants as a primary barrier to accessing these services. This study aimed to address these gaps by asking leaders from affected communities for their priorities, guidance, and advice.

### Priority areas and strategies to increase wellbeing and health outcomes

The priority needs identified for ACB communities in London included (1) mental health education, (2) ACB-tailored services, (3) a need for ACB service providers, (4) cultural safety training for staff, (5) French language services, (6) an ACB-specific role within organizations, and (7) an increase in funding and resources for these activities. Nurses, in particular, carry a moral and ethical obligation to advance health through political advocacy and are well-positioned to advocate for the prioritization of these needs, since their work intersects public policy, with the health and well-being of individuals, families, and communities (Falk-Rafael, 2005). To date, Black and other racialized nurses have been underrepresented within the Canadian healthcare system (Jefferies et al., 2019, 2022; Oudshoorn, 2020). From 2001 to 2021, there was a 130% increase in the population of racialized Canadians (Hou et al., 2023), therefore, culturally safe care must be a priority, and to achieve this, it is essential that the nursing workforce is representative of the population it serves. Amidst this nursing shortage, organizations need to do better to recruit and retain racialized nurses to the profession (Jefferies et al., 2022). To do this, the impacts of racism on the nursing workforce must be understood, as well as how nurses can engage in anti-racism work in a way that prioritizes their own cultural safety and emotional wellbeing.

#### Mental health education

Mental health issues in ACB people are prevalent due to marginalization, discrimination, being seen as a target, and living in a constant state of hypervigilance due to racism (Harris, 2021). Internalized racism also impacted the mental health of ACB communities through a process of ‘self-gaslighting’ (Johnson et al., 2021; Tobias & Joseph, 2020). Thus, this is a priority concern, as London's ACB communities experience high rates of mental health issues, but mental health is not talked about due to stigma and assumptions within the community that it is “a white people problem,” perpetuating additional harm. For example, tropes such as Black people being “strong” and not requiring help, create further barriers to accessing mental health services, or even reporting mental health concerns (Gaston et al., 2016; Patterson & Veenstra, 2016). It is important to provide culturally specific education both to ACB communities and to those providing services (Handtke et al., 2019) to improve mental health education and help eliminate these forms of stigma and discrimination. Participants suggested working with ACB communities to incorporate education on the importance of mental health in locations where these communities gather to normalize these discussions.

#### ACB-tailored services

Most of the services that currently exist in Canada follow a Eurocentric model, meaning they operate in a way that reflects the needs, beliefs, and values of European or White cultures, and White people hold the power, perpetuating systemic barriers for ACB people to access care, and not serving racialized groups in a culturally safe way (Phillips-Beck et al., 2020). There is a need for more health and social services that are tailored to the lived experiences, needs, beliefs, and values of the ACB communities. For example, the Black Healing Centre (n.d.) in Montreal launched in 2021, which offers mental health services tailored to the Black community by offering group therapy reflecting the culture's needs (instead of individual therapy- the prominent approach in Eurocentric systems reflecting individualism), providing subsidized therapy, and employing Black practitioners.

#### ACB service providers

Authentic representation of ACB communities, beyond tokenism, is required within the staff and leadership of health and social service organizations (Etowa & Hyman, 2021). ACB people do not feel comfortable or welcomed in service settings where they are not represented, resulting in alienation and a lower likelihood of accessing these services (Antabe et al., 2021). For example, many participants voiced the difficulties of confiding in a White therapist, as they felt that they would not understand their experiences. Common lived experiences and shared understanding of how being Black can impact various aspects of an individual's day-to-day life and health provide an important foundation for the therapeutic relationship for some ACB people (Meyer & Zane, 2013). Again, emphasizing the importance of having a nursing workforce that is representative of the population that it serves.

#### Cultural safety training

Cultural safety training at all levels of an organization is required to make services more welcoming and safer for ACB people (Etowa & Hyman, 2021). Once staff and leaders have a better understanding of local ACB communities and the challenges they face, they can work together to reduce and ideally abolish systemic and interpersonal racism and their impacts. Despite the need for this education, anti-racism approaches at the individual level have limited effectiveness in addressing health disparities, meaning education alone is not enough to address racism and is only a beginning step (Griffith et al., 2007).

#### French language services and navigation

The Black Francophone community in Ontario is both a linguistic and racial minority, creating additional barriers to accessing services and further marginalization (Madibbo, 2006). Navigators assist marginalized groups in mitigating the barriers that surround accessing health and social services; often the role of a professional navigator is taken on by a nurse or social worker (Rankin et al., 2022).

#### ACB-specific role

A dedicated position is needed to focus on the health of ACB communities; this role could work alongside the organization and the community to actively broker and communicate needs, and advocate for necessary programming (Clarke et al., 2013).

#### Funding and resources

Currently, there is little to no government funding for ACB-specific programs or resources within the health and social services sector; for example, LIHC would have liked to have an ACB-specific role to conduct this study, however, they had no government funding for this or ACB-specific programs or services. In 2017, the previous Government of Ontario developed an “Anti-Black Racism Strategy” that aims to address racism at the systemic level. Likewise, the Alliance for Healthier Communities (2022), the network for community primary care organizations in Ontario, created a “Black Health Strategy” to address the health disparities and barriers to accessing services that affect ACB communities. Both strategies are steps in the right direction to counter anti-Black racism, but there is still the need for government funding specific to anti-Black racism and ACB-specific services. At the local level, the Middlesex-London Health Unit (2021) has recommended funding be provided to address anti-Black racism, including for a role that focuses specifically on strengthening ACB health and creating a partnership with these communities, co-creating a strategy for engagement and providing culturally specific mental health services. Time, funding, and other resources need to be allocated for programs to support anti-Black racism in organizations both provincially and nationally.

### Self-gaslighting

People targeted by racism often dismiss microaggressions with the thought that they may be reading too much into the situation, not wanting to assume that others are acting in a racist manner. This results in the person second-guessing their initial perceptions, resulting in self-doubt. This thought process, shared by some of our participants, resembled the concept of ‘self-gaslighting’, where a person has a belief, and then they worry that others will be skeptical of this belief, resulting in believing that they were mistaken and giving up this original belief (Dandelet, 2021); i.e., discrediting their own experience. This can lead to self-devaluation, helplessness, hopelessness, and the belief that they are not deserving of care, leading people to avoid accessing care, and increased risk of adverse health outcomes (Jones, 2000). It is important to name this process so that individuals understand their experience and offset potential negative impacts (Davis & Ernst, 2019). Further research is recommended to better understand this concept in relation to the experience of racism.

### Conducting research and engaging with communities through an anti-racism Lens: ‘nothing about us – without us’

Our participants were unanimous that ACB people need to be included in any process or project that involves them, a finding consistent with the systemic review by Clarke and colleagues (2013), which identified the need for anti-racism interventions that actively engaged the community; but had only accounted for 6.5% of the anti-racism strategies they reviewed. An approach to research, program and/or policy development that is co-produced with individuals who have lived experience of the issue allows for more rich and relevant details than could be provided otherwise, helping to bridge the gap between the community and service providers, resulting in safer spaces (Rahman et al., 2022). Despite the well-known fact that the process of co-design is best practice for projects that affect specific groups or communities, it has been acknowledged that it is challenging to establish authentic relationships with communities and implementation is difficult (Jackson & Moorley, 2022).

According to our findings, to address anti-Black racism, ACB people must be involved in every step of the process and the community should be asked for their input and feedback in an iterative and authentic way (i.e., changes made and communicated, based on their feedback). For engagement to be meaningful and genuine and avoid tokenism, communities need to be involved from the conception of a project (Jackson & Moorley, 2022). Ideally, one or more members from the community is involved as a liaison throughout the work, both to inform the process and support evaluation and interpretation (Edwards et al., 2020). Including a member of the ACB community throughout the process creates a more balanced relationship and helps to eliminate the “power-over” dynamic (i.e., the power that White people and institutions hold that allows them to exert influence, control, and oppressive practices over subordinate groups) that currently exists (Madibbo, 2006). By working together and sharing power, stronger partnerships are built with improved outcomes for communities.

However, these integrated, community co-led approaches to research, practice and policy development must start by acknowledging that the responsibility of addressing anti-Black racism does not fall on Black people (Hassen et al., 2021; MLHU, 2021). The needs and experiences voiced by representatives from these communities drive solutions, but the burden, including finding and sustaining resources, must fall to the organizations and systems doing harm. Organizations need to be deliberate about their actions to address anti-Black racism and find the balance between actively and authentically involving ACB communities, while not over-burdening them. This can be done by the organization educating themselves before going to the community and asking the community for their input, rather than going to the community and expecting them to provide that basic education, which is often re-traumatizing as people are asked to share examples of the harms they have endured to prove that they are deserving of change. As we heard from participants, this is a delicate balance, but one that can be addressed through open and respectful discussion and intentional planning.

### Framework – forming authentic relationships with ACB communities

**Figure 2.**
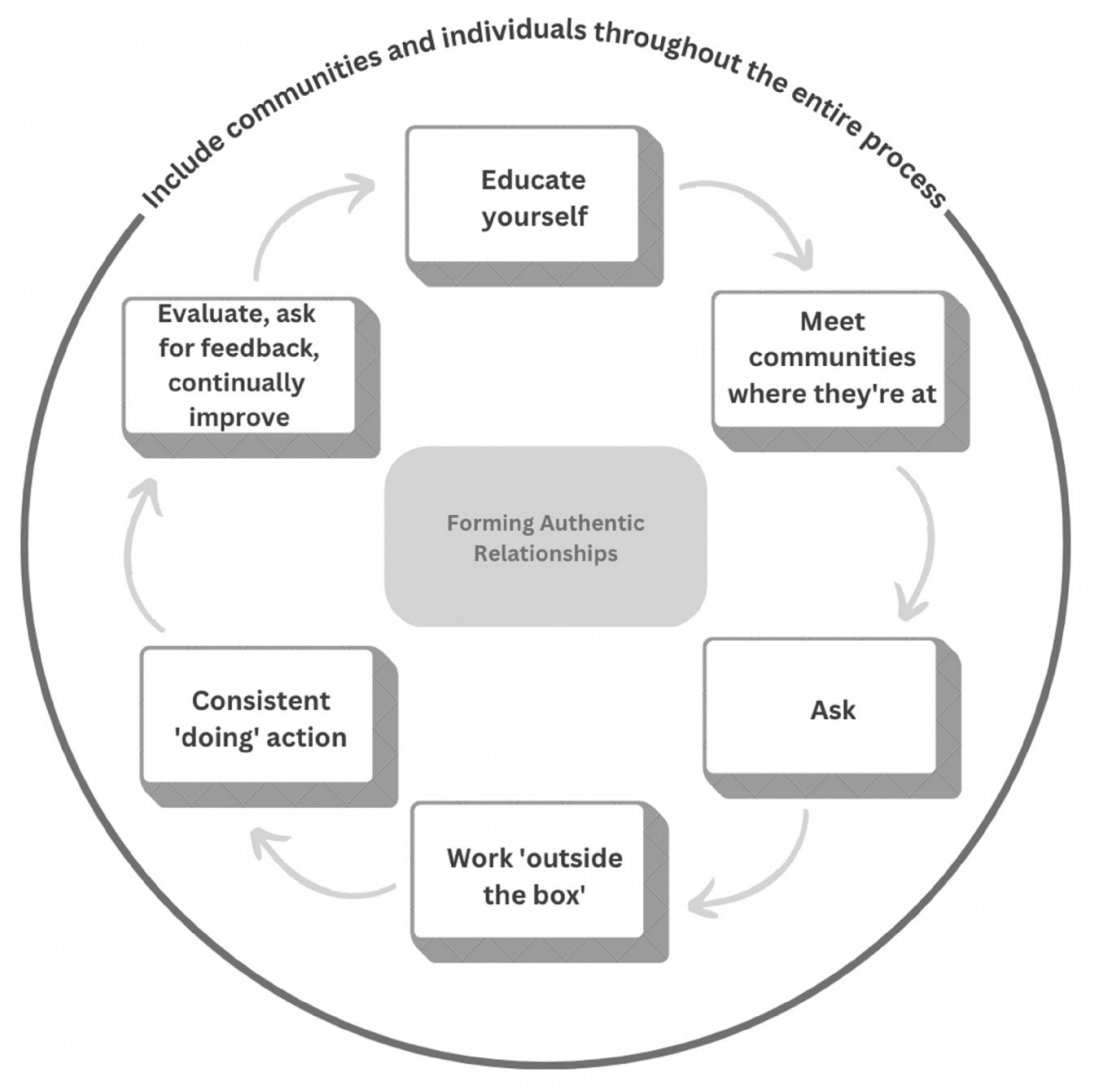
Framework for forming authentic relationships with ACB communities.

Allies are essential for successful anti-racism programs, yet there is little research on partnerships between organizations and racialized communities, and frameworks to inform anti-racism efforts for allies are underdeveloped and remain informal and unevaluated (Came & Griffith, 2018). The framework in Figure 2 was developed using our findings to assist organizations in forming authentic relationships with the ACB communities.

1. First, organizations and professionals need to *educate themselves* about local ACB communities, including histories and contexts, before engaging. Trust is essential to a successful partnership and there must be foundational knowledge to have trust (Clarke et al., 2013). The burden should not be placed on racialized communities to educate white people about racism.
2. An essential component to forming effective relationships with ACB communities is to *meet them where they are at*, meaning allowing them to determine the starting place for discussion, priority-setting, and action. This can be determined through self-education and initial consultation, especially if a liaison role has been established.
3. Next, organizations should *ask* the community what their needs are. Understanding the needs of the community is important to address anti-Black racism, and the best way to understand these needs is by hearing directly from those affected.
4. To meet the needs of the ACB communities, organizations need to *work outside the box,* meaning in non-traditional, disruptive, and innovative ways. For example, workers engaging with racialized communities need to be flexible with their work and hours to cater to the ACB communities and hold discussions in places of safety, familiarity, and comfort for community members.
5. ACB communities would like to see *consistent “doing” action*, rather than a one-time event. This is necessary to establish trust and strengthen relationships, as un-enforced policies or talk without action is performative in nature and does little to address anti-Black racism (Payton et al., 2022). These kinds of false promises can do great harm.
6. ACB communities and leaders need to be *included throughout the entire process,* as discussed in the section ‘Nothing about Us, Without Us’.
7. Finally, *evaluation*, *feedback, and continuous improvement,* based on community input, must be embedded.

### Implications and recommendations

Recommendations for anti-Black racism within organizational policies and practices were co-developed with LIHC and are summarized in Table 1.

**Table 1.** Policy and practice-focused anti-racism recommendations.

Policy-Focused Recommendations:
Organizations should incorporate anti-racism into their policies in a way that accurately describes the steps to take when racism occurs in the workplace, including safe processes for managing complaints
Include mandatory cultural safety training for staff at all levels
Focus hiring and retention practices to recruit and retain people from diverse groups
Practice-Focused Recommendations:
Create a position or role within the organization that focuses on addressing the health and well-being of ACB communities
Create awareness and availability of these services to ACB communities
Develop services that are tailored to the needs of the ACB communities by utilizing the framework for developing authentic relationships with the ACB communities

One goal of Ontario's “Anti-Black Racism Strategy” is to work with organizations that have already begun addressing anti-Black racism in the community to further inform their strategies and interventions. The results of this study could further contribute to the Ontario Government's “Anti-Black Racism Strategy”, MLHU's (2021) “Anti-Black Racism Plan”, and the Alliance for Healthier Communities’ (2022) “Black Health Strategy” by proposing a framework for organizations and institutions to form authentic relationships with ACB communities to begin to address the systemic racism that currently exists in Ontario's health and social service systems and to take action to address health inequities among ACB people.

### Limitations and future research

The purpose of this study was to gain a better understanding of the experiences and perspectives of ACB leaders on Anti-Black racism in London, Ontario. Due to the inclusion criteria, in particular, wanting to speak with community leaders, most participants were well-established in London; future research could include a broader range of ACB community members, including those more recently arrived via immigration (or from other Canadian locales) and those not involved in services or advocacy. This would also offset the potential limitation that our sample could not, and indeed was not intended to represent all potential voices across the diverse ACB communities in London.

This study was intended to begin the process of educating ourselves as researchers and as a community service provider (LIHC) regarding needs and perceptions in London's ACB communities; future work is required to expand upon this, but as noted above, must also be co-led by said communities. As we learned through our additional exploration of the issue of ‘nothing about us, without us’, we acknowledge that our study could and should have engaged a member or members of an ACB community to provide initial guidance in developing the research questions, study design and processes. In the future, a best practice would be to include a member of the community from the conception of the study (Rahman et al., 2022). A key next step for LIHC is to take the framework back to ACB communities, including service users, and “validate” it by using it to discuss needs and priority programming for co-development.

London's ACB communities are diverse, therefore engagement strategies can’t be ‘one size fits all’; it would be beneficial to have future research that explores more specifically the unique needs of sub-groups of people who identify as African, Caribbean and/or Black (or other sub-groups), including different language groups, especially Francophones. Further areas of research include advancing the understanding of self-gaslighting concerning racism and strategies to address this internalized racism and investigating the effectiveness of implementing a position focused on the health and well-being of ACB communities. To address the health disparities within the ACB communities, as well as advance the nursing profession and current Canadian nursing workforce, it will be necessary to examine the experiences of racism and anti-racism among Black nurses and how these experiences impact the recruitment and retention of racialized nurses (Jefferies et al., 2022) Our proposed framework, if implemented, will also require evaluation.

## Conclusions

Systemic racism needs to be addressed to improve the health and well-being of ACB communities in London and across Canada. Organizations and leaders within healthcare and social services must work with ACB communities, forming an authentic ‘power-with’ dynamic, to co-create programs, policies, and practices that reflect the needs of these communities.
